# Case report: Primary ovarian Burkitt's lymphoma: A puzzling scenario in pediatric population

**DOI:** 10.3389/fped.2022.1072567

**Published:** 2023-01-11

**Authors:** Giorgio Persano, Alessandro Crocoli, Cristina Martucci, Luciana Vinti, Giulia Cassanelli, Alessandra Stracuzzi, Antonello Cardoni, Alessandro Inserra

**Affiliations:** ^1^ Surgical Oncology Unit, Department of Surgery, Bambino Gesù Children’s Hospital – IRCCS, Rome, Italy; ^2^ General Surgery Unit, Department of Surgery, Bambino Gesù Children’s Hospital – IRCCS, Rome, Italy; ^3^ Department of Pediatric Hematology/Oncology and Cell and Gene Therapy, Bambino Gesù Children’s Hospital – IRCCS, Rome, Italy; ^4^ Department of Imaging, Bambino Gesù Children’s Hospital – IRCCS, Rome, Italy; ^5^ Department of Pathology, Bambino Gesù Children’s Hospital – IRCCS, Rome, Italy

**Keywords:** lymphoma, ovarian cancer, surgery, pediatrics, burkitt’s lymphoma

## Abstract

Burkitt's lymphoma (BL) is defined as a highly invasive B-cell lymphoma, usually characterized by an excellent prognosis, more than 90% of children and adolescents being cured with highly dose-intensive multiagent chemotherapy. Primary ovarian localization without involvement of other organs is a rare manifestation of BL, especially in pediatric population. Symptoms at diagnosis are similar to other ovarian lesions and differential diagnosis may be challenging for clinicians. A 12-year-old girl was referred to our institution for abdominal pain and palpable mass observed by the pediatrician. Diagnostic work-up demonstrated a large mass arising from the right ovary, causing compression on abdominal aorta, inferior vena cava, ureters and bowel, with a second smaller lesion on the left ovary. At surgery, a 15 cm-large, ruptured mass arising from the right ovary was found, associated with a second lesion originating from the left ovary (8 cm) and multiple nodules of the greater omentum. Right salpingo-oophorectomy was performed, incisional biopsies were taken from the left ovary and omental nodules and peritoneal fluid samples were collected for cytology. Pathology revealed a Burkitt lymphoma and the patient underwent chemotherapy according to AIEOP LNH-97 Protocol, group R3 with *Rituximab*. Preoperative diagnosis of primary ovarian lymphoma is extremely difficult. Surgical exploration is often necessary in patients presenting with acute abdominal or pelvic pain; when the suspicion of primary ovarian lymphoma arises intraoperatively, every effort should be made to minimize invasive procedure in order to enhance post-operative recovery.

## Introduction

Ovarian neoplasms are rare in pediatric population, accounting for approximately 1% of all childhood malignancies; among these patients, more than half are affected by germ cell tumors, followed by epithelial neoplasms and sex cord stromal tumors (1, 2). Rarely, patients presenting with isolated ovarian masses are affected by primary ovarian lymphomas (3).

Clinical presentations include abdominal or pelvic pain, dysuria and evidence of a palpable mass and, considering the overlap among the possible histotypes (3–5), differential diagnosis may be challenging for clinicians.

In the present paper, the authors report a case of an adolescent affected by primary ovarian lymphoma and discuss the problems of differential diagnosis in girls affected by ovarian neoplasms.

Ethical review and approval was not required for the study on human participants in accordance with the local legislation and institutional requirements, as well as written informed consent from the patients or participants' legal Ethical review and approval was not required for the study on human participants in accordance with the local legislation and institutional requirements, as well as written informed consent from the patients or participants' legal guardian.

## Case description

A 12-year-old girl was referred to our institution for abdominal pain and palpable mass observed by the pediatrician. After admission, a complete blood count (CBC) test was performed, showing normocytic anemia (Hb 10.3 g/dl, RBC 3.600.000/ul, mean globular volume 88.3 fl). High serum level of lactate dehydrogenase (1,219 U/ml; normal value 120–300 UI/ml) and uric acid (13.2 mg/dl; normal value 2.4–5.7 mg/dl) were reported, while the results of liver and kidney function tests were normal. Tumor markers such as Beta-human chorionic gonadotropin (β-HCG), Alfa-fetoprotein (α-FP), CA 19-9 and CEA were negative; only CA-125 was higher than the reference value (patient's value 314 UI/ml; normal value 0–35 UI/ml).

**Figure 1:**
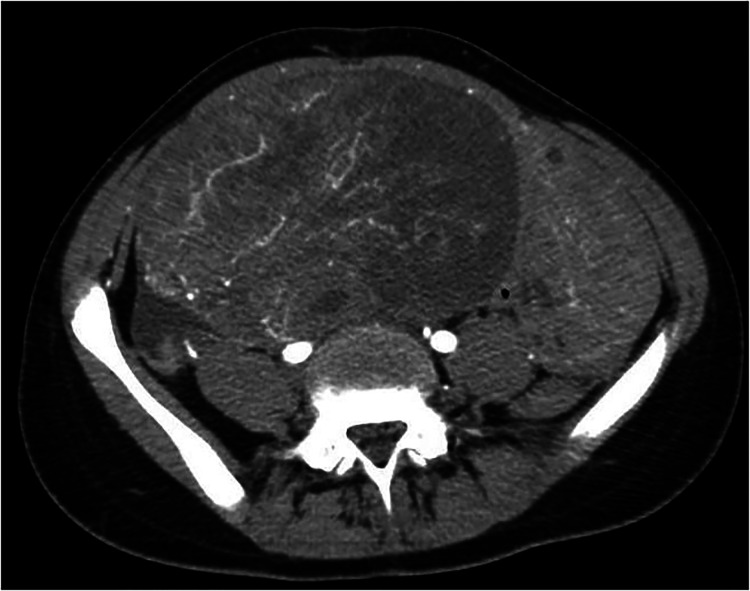
Initial CT scan.

**Figure 2:**
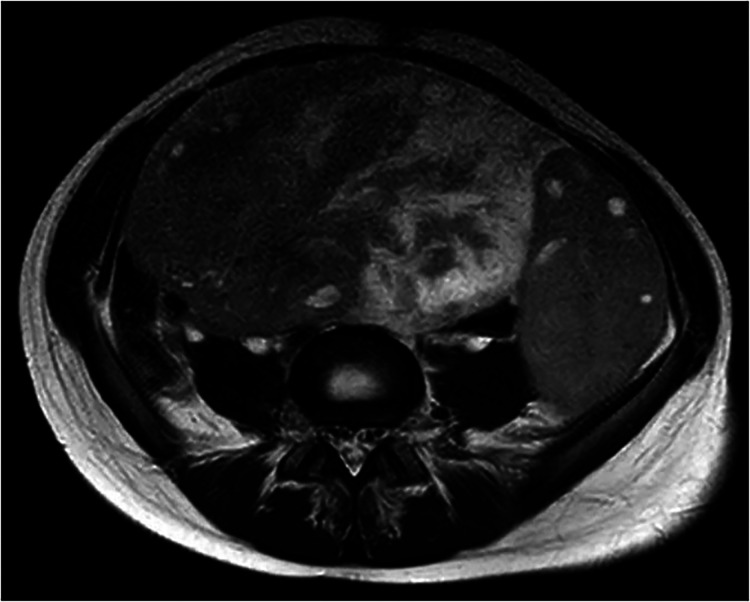
MRI images.

After initial ultrasound, the patient underwent Computed Tomography (CT) scan: the results showed a voluminous hypogenic mass (size 20 cm × 13 cm × 10 cm) arising from the right ovary, causing compression on the contiguous structures, especially abdominal aorta, inferior vena cava, ureters and bowel (Figure 1); another smaller mass (8 cm × 8 cm × 5 cm) was detected on the left ovary and abundant ascites was described. No metastasis or pathological lymph nodes were identified. Magnetic Resonance Imaging (MRI) was then performed, defining more specifically the characteristics of the neoplasm and confirming its origin from the right ovary (Figure 2).

The case was discussed in a multidisciplinary meeting with radiologists, oncologists and pathologists. Images were judged suggestive of primary right ovarian tumor with ischemic changes due to vascular compression in the contralateral ovary; surgical exploration was indicated in order to resect what was thought to be a right ovarian neoplasm and relieve compression on the left ovary.

Pfannenstiel laparotomy was performed; intraoperatively, a 15 cm-large, ruptured mass arising from the right ovary was found, associated with a second lesion originating from the left ovary (8 cm) and multiple nodules of the greater omentum. Right ovary was completely distorted by the mass and was judged non-salvageable; right salpingo-oophorectomy was then performed and incisional biopsies were taken from the left ovary and omental nodules and peritoneal fluid samples were collected for cytology. No surgical complications were reported.

**Figure 3:**
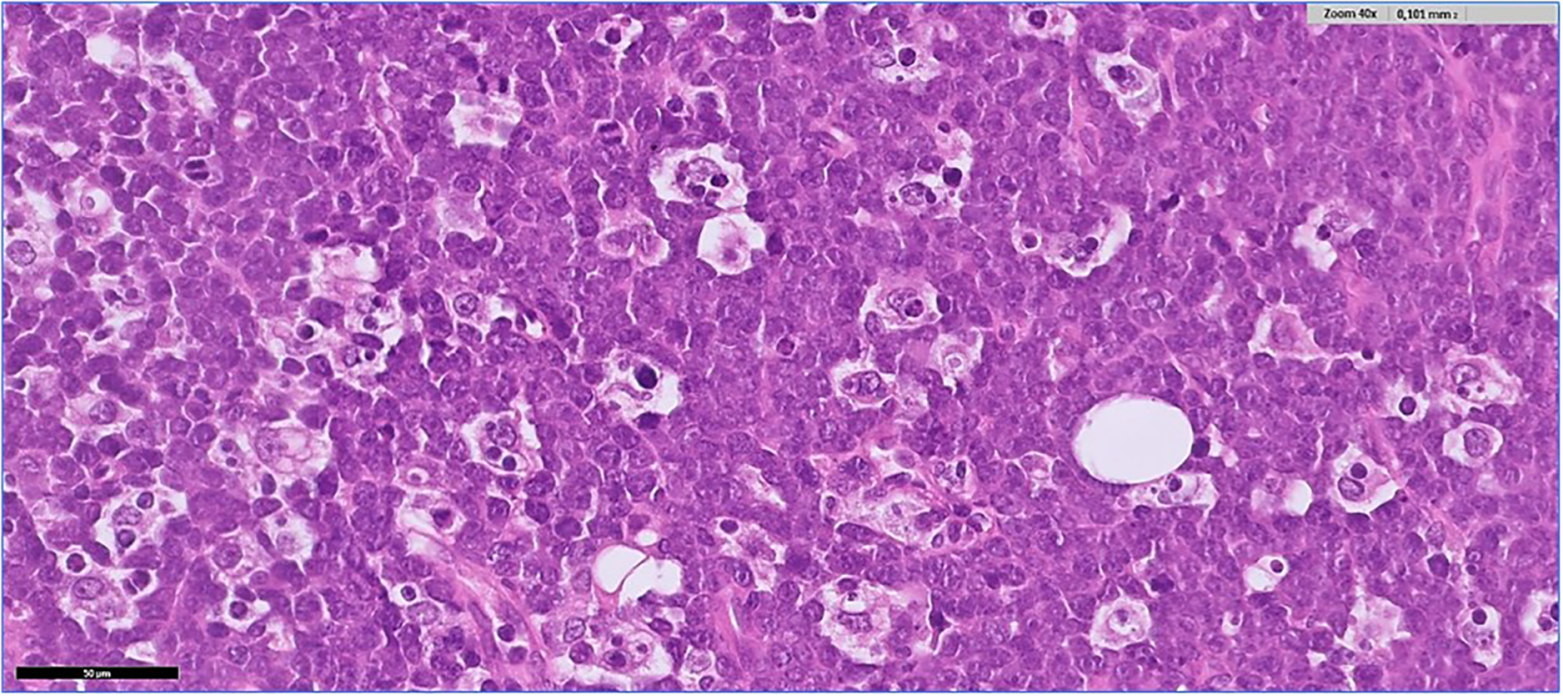
Histological aspect (Hematoxylin and Eosin stained tissue).

Histological examination revealed a diffuse proliferation of monomorphic, medium size lymphoid cells, with high nucleus/cytoplasmic ratio, finely dispersed chromatin and multiple small nucleoli. There were abundant mitoses and apoptosis and many macrophages containing tingible-bodies (“starry sky” pattern). There were also areas of necrosis (Figure 3). The same neoplastic infiltration was seen in all specimens, from both ovaries and from the omental nodules. Immunohistochemistry showed positivity of the neoplastic cells for B-cell markers (CD20, CD19, CD22, CD79a) and for germinal center B-cell markers (CD10, BCL6), and negativity for BCL2, TdT, CD138 and CD3. Proliferative index (Ki67) was positive in more than 95% of the neoplastic cells. MYC immunostain showed strong positivity in more than 90% of the neoplastic cells, suggesting a *c-myc* gene rearrangement. Fluorescence *in situ* hybridization (FISH) confirmed the presence of the MYC(8q24)-IGH(14q32) rearrangement. In situ hybridization for Epstein-Barr virus (EBER) was negative. A final diagnosis of Burkitt lymphoma was given.

The patient underwent complete staging of the disease according to the International Pediatric Non-Hodgkin Lymphoma Staging System (6) by total-body CT scan and PET-CT scan, lumbar puncture and bone marrow biopsy (both negative for neoplastic cells). At the end of the work-up the patient was classified as stage III Burkitt's lymphoma and chemotherapy according to AIEOP LNH 97 protocol, risk group R4 (7), that includes intravenous cyclophosphamide, vincristine, methotrexate, iphosphamide, cytarabine, etoposide, daunorubicin and dexamethasone and intrathecal therapy with methotrexate, cytarabine and methylprednisolone, was started five days after surgery, associated with intravenous Rituximab.

At the time of writing, the patient is still under induction chemotherapy (V course), which has been, so far, well tolerated.

**Figure 4:**
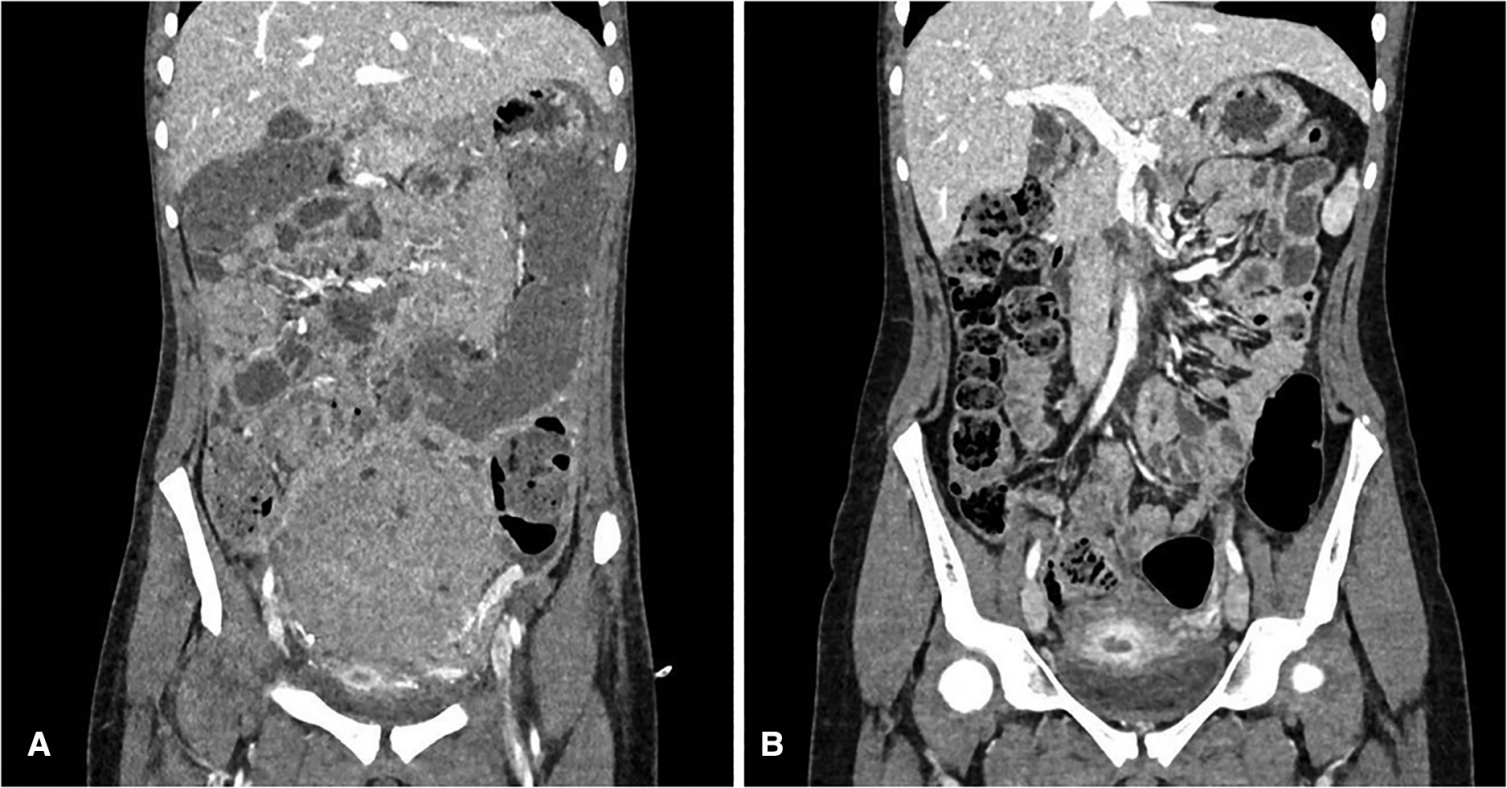
(**A**) CT scan after surgery (before chemotherapy); (**B**) CT scan after four cycles of chemotherapy.

Follow-up imaging shows reduction of the mass in the left ovary (Figure 4).

## Discussion

The incidence of ovarian tumors in girls and adolescents increases with age, starting from 0.4 per 100,000 during infancy to 25–30 per 100,000 at the age of 18 (8); they are mainly represented by benign lesions (80% of cases) (4). Primary lymphomas of the ovary are rare findings, accounting for less than 1% (9), and may appear as a localized ovarian lesion or as aggressive and metastatic form, frequently involving the central nervous system (CNS) and bone marrow (3). The sporadic type is frequently seen in patients under the age of 35, with a higher incidence among Caucasians and Central Americans (10).

Symptoms at presentation are very similar across different histologic types and include abdominal distension, palpable pelvic masses or acute pain due to torsion or rupture (3–5, 11). Abnormal vaginal bleeding, irregular menses, urination, bowel obstruction, ascites, fever, nights sweats, fatigue and weight loss are possible additional symptoms. Meningeal infiltration, headaches, visual impartment, and paraplegia may occur if the CNS is affected (3). When systemic symptoms are absent, differential diagnosis is impossible on clinical presentation alone.

The most prevalent ovarian lymphoma imaging characteristics on CT scan, ultrasonography, and MRI are described by Ferrozzi et al. (12) When compared to ultrasound, which has homogeneous, hypoechogenic structures and a non-specific appearance, CT scans show hypodense lesions with modest contrast enhancement. Hypointense T1-weighted scans and slightly hyperintense T2-weighted images on an MRI reveal homogenous masses with areas of contrast enhancement, irregular margins, extracapsular tumor growth and ascites (13); however, different malignant histotypes might have similar appearance on MRI (14). It's interesting to note that bilateral ovarian involvement was seen in 67% of primary ovarian lymphoma cases reported (3); this feature is highly suggestive but not exclusive for ovarian lymphoma, since bilateral lesions can occasionally be observed both in ovarian germ cell tumors (15) and in epithelial neoplasms (5). Ovarian lymphoma is the most likely diagnosis if ascites is absent and a homogenous bilateral tumor occurs in the ovaries.

Serum levels of carcinoembryonic antigen (CEA), carbohydrate antigen 19-9 (CA19-9), alpha-fetoprotein (AFP), and β-HCG biomarkers usually remain normal in patients with ovarian lymphoma but may be helpful for the differential diagnosis of other ovarian tumors. Serum levels of LDH and uric acid are usually elevated in patients affected by Non-Hodgkin lymphomas (16) and high levels of CA-125 have been associated with primary ovarian lymphomas (3). These serum markers may help differentiate between benign and malignant lesions, although they are not specific; high levels of serum LDH can be observed in advanced-stage germ cell tumors or in epithelial tumors (5, 17), while high level of CA-125 might be suggestive of epithelial tumors (5) or endometriosis (18).

Staging is crucial in assessing the extent of the disease and enables the application of a standardized treatment strategy; in the pediatric population, the St. Jude staging system has largely been replaced with the International Pediatric Non-Hodgkin Lymphoma Staging System (6).

Treatment of primary lymphoma of the ovary consists in multiagent chemotherapy (3, 7). Surgical procedures in ovarian lymphoma play a role in the diagnostic process, providing samples for diagnosis and staging(3); extensive debulking or bilateral oophorectomy is not beneficial and tumor resection should be avoided since recovery from surgery may delay the initiation of systemic therapy, which is needed urgently in this fast-growing tumor (19, 20).

Differentiating ovarian lymphoma from other ovarian neoplasms is essential, since ovarian lymphoma is treated by systemic chemotherapy (3, 6) while in germ cell tumors and epithelial neoplasms surgical resection is indicated (5, 11), followed by chemotherapy only in advanced stage disease (4, 15).

Despite the limited role of surgery in ovarian lymphoma, the majority of reported patients underwent laparotomic or laparoscopic exploration on the basis of the acute clinical presentation and the suspicion of adnexal torsion (3, 21, 22); in the present case, the patient complained of lower abdominal pain that was interpreted as a sign of vascular compression, and surgical exploration was therefore warranted.

Although lymphomas have a rapid growth rate, the estimated survival rate in pediatric patients is greater than 90%, while lower values are reported in prospective clinical trials for adults, ranging 75%–85% (23–25). Young patients with lymphomas who receive aggressive therapies have excellent outcomes, but treatment-related toxicity is still a considerable challenge in the treatment of lymphoma, especially in older patients. In adult patients, the five-year survival rate in advanced-stage disease has been reported as 60%–85% (26); in pediatric and adolescent population there is little information available regarding long-term prognosis of lymphomas involving the ovary.

In conclusion, preoperative diagnosis of primary ovarian lymphoma is extremely difficult. Laboratory tests including a complete blood count, comprehensive metabolic panels, measurement of LDH and uric acid levels can be helpful in the differential diagnosis of ovarian neoplasms.

Surgical exploration is often necessary in patients presenting with acute abdominal or pelvic pain; when the suspicion of primary ovarian lymphoma arises intraoperatively, every effort should be made to minimize invasive procedure in order to enhance post-operative recovery.

**Timeframe of the relevant events**

**Table:** 

Time	March 2022	April 2022	24th April 2022	26th April 2022	27th April 2022	28th April 2022	29th April 2022	4th May 2022
Event	Referred By Pediatrician For Abdominal Pain And Increasing Abdominal Girth	Ultrasound Showing Pelvic Mass	Ct Scan	Mri	Referred To Our Institution	Lab Tests:	Surgical Excision	Chemotherapy And Immunotherapy Start
						Ldh: 1,219 U/ml (normal value 120–300 UI/ml)		
						Uric Acid: 13.2 mg/dl (normal value 2.4–5.7 mg/dl)		
						Creatinine: 0.64 mg/dl (normal value 0.44–0.68 mg/dl)		
						Calcium: 8.4 mg/dl (normal value 8.4–10.2 mg/dl)		
						Phosphate: 3.2 mg/dl (normal value 2.8–4.8 mg/dl)		
						Potassium: 4.1 Meq/L (normal value 3.1–5.1 Meq/L)		

## Data Availability

The original contributions presented in the study are included in the article/Supplementary Material, further inquiries can be directed to the corresponding author.
